# Human CD4^+^ T cell subsets differ in their abilities to cross endothelial and epithelial brain barriers in vitro

**DOI:** 10.1186/s12987-019-0165-2

**Published:** 2020-02-03

**Authors:** Hideaki Nishihara, Sasha Soldati, Adrien Mossu, Maria Rosito, Henriette Rudolph, William A. Muller, Daniela Latorre, Federica Sallusto, Mireia Sospedra, Roland Martin, Hiroshi Ishikawa, Tobias Tenenbaum, Horst Schroten, Fabien Gosselet, Britta Engelhardt

**Affiliations:** 1 Theodor Kocher Institute, University of Bern, Bern, Switzerland; 2 Department of Pediatrics, Pediatric Infectious Diseases, Medical Faculty Mannheim, Heidelberg University, Mannheim, Germany; 3 Feinberg School of Medicine, Northwestern University, Chicago, IL USA; 4 Institute for Research in Biomedicine, Università Della Svizzera Italiana, Bellinzona, Switzerland; 5 Institute for Microbiology, ETH Zurich, Zurich, Switzerland; 6 Neuroimmunology and MS Research Section (NIMS), Neurology Clinic, University of Zurich, University Hospital Zurich, Zurich, Switzerland; 7 Laboratory of Clinical Regenerative Medicine, Department of Neurosurgery, Faculty of Medicine, University of Tsukuba, Tsukuba, Japan; 8 Blood Brain Barrier Laboratory, University of Artois, Lens, France; 9 Present Address: Transcure Bioservices, Archamps, France; 10 Present Address: Center for Life Nanoscience, Istituto Italiano di Tecnologia, Rome, Italy

**Keywords:** Blood–brain barrier, Blood-cerebrospinal fluid barrier, T-cell migration, Adhesion molecule, Multiple sclerosis

## Abstract

**Background:**

The brain barriers establish compartments in the central nervous system (CNS) that significantly differ in their communication with the peripheral immune system. In this function they strictly control T-cell entry into the CNS. T cells can reach the CNS by either crossing the endothelial blood–brain barrier (BBB) or the epithelial blood-cerebrospinal fluid barrier (BCSFB) of the choroid plexus (ChP).

**Objective:**

Analysis of the cellular and molecular mechanisms involved in the migration of different human CD4^+^ T-cell subsets across the BBB versus the BCSFB.

**Methods:**

Human in vitro models of the BBB and BCSFB were employed to study the migration of circulating and CNS-entry experienced CD4^+^ T helper cell subsets (Th1, Th1*, Th2, Th17) across the BBB and BCSFB under inflammatory and non-inflammatory conditions in vitro.

**Results:**

While under non-inflammatory conditions Th1* and Th1 cells preferentially crossed the BBB, under inflammatory conditions the migration rate of all Th subsets across the BBB was comparable. The migration of all Th subsets across the BCSFB from the same donor was 10- to 20-fold lower when compared to their migration across the BBB. Interestingly, Th17 cells preferentially crossed the BCSFB under both, non-inflamed and inflamed conditions. Barrier-crossing experienced Th cells sorted from CSF of MS patients showed migratory characteristics indistinguishable from those of circulating Th cells of healthy donors. All Th cell subsets could additionally cross the BCSFB from the CSF to ChP stroma side. T-cell migration across the BCSFB involved epithelial ICAM-1 irrespective of the direction of migration.

**Conclusions:**

Our observations underscore that different Th subsets may use different anatomical routes to enter the CNS during immune surveillance versus neuroinflammation with the BCSFB establishing a tighter barrier for T-cell entry into the CNS compared to the BBB. In addition, CNS-entry experienced Th cell subsets isolated from the CSF of MS patients do not show an increased ability to cross the brain barriers when compared to circulating Th cell subsets from healthy donors underscoring the active role of the brain barriers in controlling T-cell entry into the CNS. Also we identify ICAM-1 to mediate T cell migration across the BCSFB.

## Background

Central nervous system (CNS) homeostasis is guaranteed by the endothelial, epithelial and glial brain barriers. The endothelial blood–brain barrier (BBB) is localized to the wall of small CNS blood vessels. The epithelial blood cerebrospinal fluid barrier (BCSFB) is surrounding the choroid plexuses localized in all brain ventricles. Last but not least the glia limitans composed by the parenchymal basement membrane and astrocyte end feet is surrounding the entire CNS parenchyma at the surface (glia limitans superficialis) and towards the blood vessels (glia limitans perivascularis) [1].

The brain barriers protect the CNS from the changing milieu of the blood stream but also strictly control immune surveillance of the CNS [2]. Brain barriers breakdown and uncontrolled immune cell infiltration into the CNS are early hallmarks of multiple sclerosis (MS), the most common neuroinflammatory disorder in young adults that can lead to severe disability. Immune cell infiltration across the BBB is tightly regulated by the sequential interaction of adhesion or signaling molecules on immune cells and the BBB endothelium [3]. Less is known about the mechanisms regulating immune cell migration across the BCSFB. Current knowledge about the molecular mechanisms mediating immune cell trafficking across brain barriers are mainly derived from experimental autoimmune encephalitis (EAE) (reviewed in [3]), an animal model of MS. EAE has allowed to develop efficient therapies targeting immune cell trafficking across the BBB for the treatment of relapsing–remitting MS (RRMS) [4]. Unfortunately these therapies are associated with progressive multifocal leukoencephalopathy (PML) caused by the infection of CNS cells with the JC virus [5]. This suggests that the current therapeutic strategies besides successfully inhibiting the migration of pathogenic immune cells into the CNS also interfere with CNS immune surveillance. This underscores the urgent need to improve our understanding of the anatomical routes and molecular mechanisms used by different immune cell subsets to enter the CNS.

While the etiology of MS remains unknown recent genome-wide association studies (GWASs) underscored the involvement of CD4^+^ T helper (Th) cells in MS pathogenesis [6, 7]. CD4^+^ T cells are divided into several subsets, which are defined by lineage-specifying transcription factors, expression of signature cytokines and distinct chemokine receptors allowing these T cells to exert different effector functions and to migrate to different tissues. For instance, Th1 cells express T-bet, secrete IFN-γ, allowing them to help macrophages to eliminate intracellular viruses and bacteria, and preferentially express CXCR3; Th2 cells express GATA-3, produce IL-4, IL-5, and IL-13, which are relevant for eliminating extracellular parasites, and preferentially express CCR3 and CCR4; classical Th17 cells express RORγt, produce IL-17A, IL-17F, and IL-22, making them efficient helpers for eliminating extracellular bacteria and fungi, and preferentially express CCR6 [8]. The CCR6^+^ Th cell subset comprises also cells producing IFN-γ or IFN-γ and IL-17, defined as Th1* [8, 9].

Th1, Th17, and Th1* cells have been suggested to be involved in MS pathogenesis. However, the degrees of their disease involvement as well as the cellular and molecular mechanisms they use to enter the CNS remain incompletely understood. IFNγ and IL-17 are elevated in the CSF of MS patients, especially during the active phase of the disease, and are also found in the CNS parenchyma of post-mortem tissue of MS patients [10–12]. Besides Th1 cells and Th17 cells, Th1* cells (expressing both T-bet and RORγt, and CXCR3 and CCR6 [13]) are found in the CSF during early disease, in post-mortem MS brain tissues [9, 12, 14] and in autoproliferating T cells, which are enriched for brain-homing CD4^+^ T cells [15]. Interestingly the Th17/Th1 ratio of CNS infiltrating cells determines lesion localization within the CNS in the EAE model [16, 17], suggesting that these different effector T-cell subsets may enter the CNS via different pathways resulting in different localization of the CNS lesions. In fact, the different CD4^+^ T cell subsets express characteristic sets of chemokine receptors (Th1: CXCR3^+^, Th1*: CXCR3^+^, CCR6^+^, Th2: CCR4^+^, Th17 CCR6^+^, CCR4^+^), which may allow them to use different anatomical routes for CNS entry. Observations in EAE [18–21] have shown that Th17 cells preferentially enter the brain via the choroid plexus in a CCR6/CCL20 dependent manner [19] and require lymphocyte function-associated antigen 1 (LFA-1) but not α4-integrins [18]. In contrast, Th1 cells preferentially infiltrate the spinal cord by crossing the BBB using α4β1-integrins [20, 21]. If the different abilities of mouse Th1 and Th17 to cross the BBB versus the BCSFB translates to human Th cell subsets has not been explored.

To investigate if different human CD4^+^ Th subsets display different abilities to cross the BBB versus the BCSFB during CNS immune surveillance and neuroinflammation, we here employed human stem cell-derived brain-like endothelial cells (BLECs) and a human choroid plexus papilloma cell line (HIBCPP) as in vitro models of the BBB and BCSFB, respectively. Both BLECs and HIBCPP cells have previously been shown to phenocopy characteristics of a functional BBB and BCSFB, respectively. BLECs and HIBCPP form mature adherens and tight junctions, show low permeability to small molecular tracers, establish high electrical resistance, show functional expression of characteristic transporters and efflux pumps and display apical/basolateral polarity [22–31]. In addition, both human brain barrier models were previously shown by us and others to show cytokine inducible expression of adhesion molecules and to be suitable for studying immune cell trafficking across the BBB and BCSFB in vitro [26, 28, 32–34]. Using different Th subsets isolated from the blood of healthy donors or from the CSF of MS patients, we directly compared the migration of circulating and CNS entry experienced Th1, Th1*, Th2 and Th17 cells across BLECs and HIBCPP monolayers under inflammatory or non-inflammatory conditions in vitro. Our data underscore that different Th-cell subsets use different cellular and molecular cues to cross the BBB and BCSFB and that neuroinflammation will impact on these mechanisms. Understanding Th-cell subset specific mechanisms of CNS entry bears the hope for developing safer therapies that specifically block the migration of pathogenic T cells into the CNS, while leaving the migration of T cells responsible for CNS immune surveillance unaffected.

## Material and methods

### Human in vitro BBB model

The French Ministry of Higher Education and Research approved the protocol regarding the use of human tissues and cells (CODECOH Number DC2011-1321). All patients gave their consents. BLECs were used as a human in vitro BBB model exactly as described before [22, 28, 32]. In brief, CD34^+^ cells were isolated from human umbilical cord blood and differentiated to endothelial cells in ECM basal medium (ScienCell) supplemented with 20% (v/v) fetal bovine serum (FBS; Life Technologies) and 50 ng/mL of VEGF_165_ (PeproTech Inc.). To induce a BBB phenotype, CD34^+^ cell-derived endothelial cells were cultured on Matrigel-coated filter inserts (PC membrane, pore size 3.0 μm; Costar, 3402) for 7 days and then co-cultured with bovine pericytes at bottom for 6 days allowing them to differentiate into brain-like endothelial cells (BLECs) as described [32]. For transendothelial electrical resistance (TEER) measurements, CD34^+^ cell-derived endothelial cells were cultured on 0.4 μm pore filter (PC membrane, Costar, 3401) and start co-culture with bovine pericytes at the same day. TEER was measured using a Volt-Ohm-Meter (Millicell ERS-2, MERSSTX01-electrode). In order to calculate the net resistance in Ω × cm^2^ of the cell monolayers, TEER value of an empty filter was subtracted from each measurement and TEER values in Ohm were multiplied by the surface area of the filters (1.12 cm^2^) as follows. TEER (Ohm × cm^2^) = (cell monolayer resistance − empty Transwell filter resistance) × surface area (cm^2^).

### Human in vitro BCSFB model

Approval was obtained by the local ethics committee of the Medical Faculty of Mannheim, Heidelberg University (2009-327N-MA). HIBCPP cells derived from a human choroid plexus papilloma were used as a human in vitro BCSFB model as described [26, 34]. In brief, HIBCPP cells were cultured in a T75 flask using HIBCPP medium (DMEM/F12 (1:1) supplied with 15 mM HEPES (Gibco), 4 mM l-Glutamine (Gibco), 1% Penicillin and Streptomycin (Gibco), 0.05% human Insulin solution (Sigma-Aldrich), and 10% heat inactivated fetal bovine serum). Once HIBCPP cells reached 80% confluency, cells were ready to be used for starting either the inverted or standard culture models of the human in vitro BCSFB model. Medium was changed every two days and only HIBCPP cells between passages 21 and 38 were employed.

For inverted culture models, HIBCPP cells were seeded on non-coated inverted Millicell^®^ filters (pore size 5.0 μm, pore density 2.0 × 10^6^ pores per cm^2^, growth area 0.33 cm^2^; Millicell, MCMP24H48) with a density of 1.0 × 10^5^ cells/mL (day 0). Twenty-four hours after seeding (day 1) Millicell^®^ filters were inverted and HIBCPP medium was added to both upper and bottom compartments. Transepithelial electric resistance (TEER) was measured for each Millicell^®^ filter from day 3 until day 6 as described above. When TEER values increased above 70 Ω × cm^2^, culturing medium was changed to HIBCPP medium containing 1% FBS allowing to improve barrier characteristics, as previously described [23, 34]. HIBCPP monolayers were used for permeability or transmigration assays at day 6 when TEER values were ≥ 500 Ω × cm^2^.

For standard culture models, HIBCPP cells were seeded on the upper part of the Millicell^®^ filters (Millicell #MCMP24H48) with a density of 1.7 × 10^5^ cells/mL (day 0). Twenty-four hours after seeding (day 1) HIBCPP medium with 10% FBS was exchanged and added respectively, on the upper and bottom compartment. The following steps from day 3 to day 6 of the standard culture model were identical to the inverted culture model.

### Investigation of cell surface expression of adhesion molecules by flow cytometry

BLECs were cultured on 3 μm pore filter (PC membrane, Costar, 3402) as described above and before [32]. HIBCPP cells were cultured with HIBCPP-medium with 10% FBS in T12.5 flasks at 37 °C (5% CO_2_) until reaching 80% confluency. BLECs and HIBCPP cells were stimulated or not prior to the experiment with 1 ng/mL of recombinant human TNF-α (R&D systems, 210TA) and 20 IU/mL recombinant human IFN-γ (R&D systems, 285IF) for 16 h at 37 °C (5% CO_2_). After stimulation, cells were washed once with HBSS supplied with 25 nM HEPES and gently detached with Accutase (Innovative cell technology) at 37 °C (5% CO_2_). Cells were washed and resuspended in FACS-buffer (DPBS (1×), 2.5% FBS, 1% NaN_3_). Then 2 × 10^4^ cells per well were transferred to a 96-well microtiter plate and then incubated 20 min on ice with the fluorochrome-conjugated antibodies or respective isotype controls (Table 1). After staining, cells were washed twice with DPBS and measured with an Attune NxT Flow Cytometer (Thermofisher Scientific, Switzerland). Data were analyzed using FlowJoTM 10 software (Tree Star, Ashland, OR, USA).

**Table 1:** Fluorophore labelled antibodies and isotype controls for FACS analysis

Antibodies	Fluorophore	Clone	Source	Cat. N.	Isotype
Anti-human ICAM1	BV421	HA58	BD Biosciences	564077	m-IgG1, κ
Anti-human ICAM2	PE	CBR-IC2/2	BD Biosciences	558080	m-IgG2a, κ
Anti-human VCAM1	FITC	51-10C9	BD Biosciences	551146	m-IgG1, κ
Anti-human P-selectin	BV510	AC1.2	BD Biosciences	743756	m-IgG1, κ
Anti-human E-selectin	APC	68-5H11	BD Biosciences	551144	m-IgG1, κ
Anti-human CD99	PE-Cy7	3B2/TA8	Biolegend	371314	m-IgG2a, κ

Isotype controls	Fluorophore	Clone	Source	Cat. N.	
m-IgG1, κ	APC	MOPC-21	BD Biosciences	555751	
m-IgG1, κ	BV421	X40	BD Biosciences	562438	
m-IgG2a, κ	PE	eBM2a	Invitrogen	12-4724-42	
m-IgG1, κ	FITC	MOPC-21	Biolegend	400108	
m-IgG1, κ	BV510	X40	BD Biosciences	562946	
m-IgG2a, κ	PE-Cy7	MOPC-173	BIolegend	400232	

### Immunofluorescence stainings

Live BLECs and HIBCPP cells were incubated with 10 µg/mL of antibodies against intercellular adhesion molecule-1 (ICAM-1, R&D System, clone BBIG, BBA3), ICAM-2 (FITZGERALD, clone CBR-IC2/2, 10R-7606), or vascular cell adhesion molecule-1 (VCAM-1, R&D system, AF809), for 20 min at 37 °C. After washing twice with DPBS, cells were fixed in 1% (w/v) formaldehyde and permeabilized in Triton X-100 (0.1% (w/v) at room temperature (RT). Then cell monolayers were blocked for 10 min with skimmed milk 5% (w/v) in PBS. For the staining of P-selectin, E-selectin, or CD99, BLECs and HIBCPP cells were first fixed with 1% (w/v) formaldehyde and permeabilized in Triton X-100 (0.1% (w/v) at RT. BLECs or HIBCPP cells monolayers were then incubated with antibodies against P-selectin (Santa Cruz, SC-19996), E-selectin (BioLegend, clone HAE-1f, 336002), or CD99 (Hec2, [35]) for 1 h at RT. After washing, fluorescently labeled secondary antibodies (Cy™3 AffiniPure Donkey Anti-Mouse IgG (H + L), Jackson ImmunoResearch) were incubated for 1 h at RT. Nuclei were stained with DAPI at 1 µg/mL. After three steps of washing with DPBS, cell monolayers on filters were mounted with Mowiol (Sigma-Aldrich) and analyzed with a Nikon Eclipse E600 microscope using the Nikon NIS-Elements BR3.10 software (Nikon, Egg, Switzerland).

### Permeability (Pe) assay

Permeability of BLEC and HIBCPP monolayers was assessed by measuring the clearance of Lucifer Yellow (LY, Lucifer Yellow CH dilithium salt, 457.25 Da, Sigma-Aldrich) as previously described [22, 36–38]. Briefly, BLECs and HIBCPP were stimulated with 1 ng/mL TNF-α and 20 IU/mL IFN-γ for 16 h prior to the measurement. LY added to the upper compartment of the filter inserts at a concentration of 50 μM. For the clearance experiments, the amount of fluorescent tracer diffusing across the monolayers was collected from bottom well every 20 min for a total of 60 min, and fluorescence intensity was measured in a Tecan Infinite M1000 multi-well reader (Tecan Trading AG). The clearance principle was used to calculate the permeability coefficient (Pe) and to obtain a concentration-independent transport parameter as previously described in detail [37]. The experiments were done in triplicates for each condition.

### Human Th cell subsets

Human CD4^+^ T cells were isolated from buffy coats of healthy blood donors obtained from the Swiss Red Cross. Human primary cell protocols were approved by the Swiss Federal Office of Public Health (authorisation N. A000197/2 to F. Sallusto). Informed consent from blood donors was approved by the local ethical committee (Comitato Etico Cantonale, http://www.ti.ch/CE, authorization n. CE3428). CD4^+^CD45RO^+^ T-helper (Th1, Th1*, Th2 and Th17) cells were isolated as previously described [39, 40] by fluorescence activated cell sorting according to their specific expression pattern of chemokine receptors (CXCR3^+^CCR4^−^CCR6^−^ for Th1; CXCR3^+^CCR4^−^CCR6^+^ for Th1*, CCR4^+^CXCR3^−^CCR6^−^ for Th2; CCR6^+^CCR4^+^CXCR3^−^ for Th17) from the peripheral blood of healthy donors and from the CSF of relapsing-remitting MS patients (Table 2). T cells were expanded for 20 days with periodic re-stimulation with 1 μg/mL phytohaemagglutinin, irradiated allogeneic peripheral blood mononuclear cells, and human interleukin 2 (IL-2, 500 IU/mL) as previously described [39–41]. This methodology has proven to allow to keep the effector T cells in culture for a maximum of 4 weeks, when their viability is reduced [41]. In the present study after 20 days of expansion, T cells were frozen and stored in liquid nitrogen until employed in the experiments. Their stable polarization was confirmed by flow cytometry analysis for the respective chemokine receptors and signature cytokines and the respective signature cytokines: IFNγ for Th1; IFNγ + IL-17 for Th1*; IL-4 for Th2 and IL-17 for Th17 [41]. Previous studies have proven suitability of these human T cell subsets in studying their biological functions including their migration properties [15, 28, 32, 39, 42–46]. T cells were thawed 1 day prior to the respective experiment and labeled with 1 μM CellTracker™ Green (CMFDA Dye, Life technologies) at 37 °C (5% CO_2_) for 30 min at the day of the experiment. After labelling, T cells were washed and dead cells were removed by Ficoll-Hypaque gradient (780 g, 20 min, 20 °C). T cells were washed twice and resuspended in migration assay medium (DMEM, 5% FBS, 4 mM l-Glutamine, 25 mM HEPES) in the appropriate concentration.

**Table 2:** Characteristics of the CSF samples from MS patients

Donor	Type	Gender	Age	Disease duration (month)	Treatment before CSF	CSF (cells/μL)	Total Protein CSF (mg/L)	IgG index	Oligoclonal band	Clinical disease activity	MRI Gd enhancement
P-1	CIS	F	19	0	–	2	233	0.50	+	+	−
P-2	RRMS	F	25	10	–	6	470	0.64	+	−	−
P-3	RRMS	F	33	2	–	7	338	1.96	+	+	+
P-4	RRMS	M	50	34	–	3	486	0.98	+	−	+
P-5	RRMS	M	22	13	–	7	263	0.52	+	+	+

### Intrinsic T-cell migration behavior

Intrinsic T-cell migration behavior was assessed by letting T cells migrate for 2 h across laminin (from Engelbreth-Holm-Swarm murine sarcoma basement membrane, Sigma) coated Millicell^®^ filters (pore size 5.0 μm, pore density 2.0 × 10^6^ pores per cm^2^, growth area 0.33 cm^2^, Millicell, MCMP24H48). In brief, filters were coated with 50 μg/mL laminin diluted in DPBS (1×) for 30 min at RT and let the filter dry for 60 min at RT. 1.5 × 10^5^ T cells/well were added to the top compartment of the Millicell^®^ filters and allowed to migrate for 2 h at 37 °C (10% CO_2_). Migrated T cells were collected from the bottom compartment and counted with an Attune NxT Flow Cytometer by gating on CMFDA positive cells.

### Transmigration assay

T-cell transmigration assay across BLECs and HIBCPP cells was performed exactly as previously described [32]. In brief, BLECs and HIBCPP cells were stimulated with both 1 ng/mL TNF-α and 20 IU/mL IFN-γ in the serum-containing culture medium for 16 h. 1.5 × 10^5^ labeled T helper cells (either Th1, Th1^∗^, Th2, or Th17 cells) were added to the upper compartment and allowed to cross BLECs or HIBCPP cells monolayer for 8 h at 37 °C (10% CO_2_). After 8 h transmigration, T cells were collected from the bottom compartment and counted with the Attune NxT Flow Cytometer by gating on CMFDA positive cells. Each experiment was performed in triplicates for each condition. When using function blocking antibodies, HIBCPP cells were pre-incubated with either anti-human ICAM-1 (10 μg/mL; clone BBIG-I1 (11C81), R&D Systems, apical side of HIBCPP cells of both inverted and standard culture models) or anti-human CD99 (20 μg/mL; clone hec2 [35], basolateral side of HIBCPP cells of the inverted culture model), or appropriate isotype control antibody for 30 min at 37 °C (10% CO_2_) before starting the TMA. T helper cells from three healthy donor and five MS patients were used in assays at least triplicate in each condition. In case the number of cells were insufficient, only 2–3 samples were involved.

### Adhesion cell counts after transmigration assay

After transmigration assay, filters were washed twice with warmed HBSS and fixed with 37% formaldehyde vapor for 2 h at room temperature. Filters were then washed twice with DPBS and blocked and stained as described above. Antibody against VE-Cadherin (Santa Cruz, clone F-8, sc-9989) was used for checking the confluent BLECs monolayer after transmigration assay. Fluorescence labeled Th cells bound per pre-defined field of view (FOV) were analyzed by fluorescence microscopy (Nikon Eclipse E600) and FIJI software (Version 2.0.0, Image J, USA). Adhesion cells/FOV were determined by mean of counting two fields per filter. Assays were performed in at least triplicates for each condition.

### Statistical analysis

Statistical analyses comprising calculation of degrees of freedom were done using GraphPad Prism 7 software (Graphpad software, La Jolla, CA, USA). Data are shown as the mean ± SD with 95% confidence interval (p < 0.05*, p < 0.01**, p < 0.001***, p < 0.0001****). To compare two groups, statistical significance was assessed by unpaired t-test, while more groups were analyzed by one-way ANOVA followed by Tukey’s multiple comparison test or two-way ANOVA followed by Tukey’s or Sidak’s multiple comparison test. The respective statistical methodology used for each assay is specified in corresponding figure legends.

## Results

### Cell surface expression of adhesion molecules on BLECs and HIBCPP cells

**Fig. 1:**
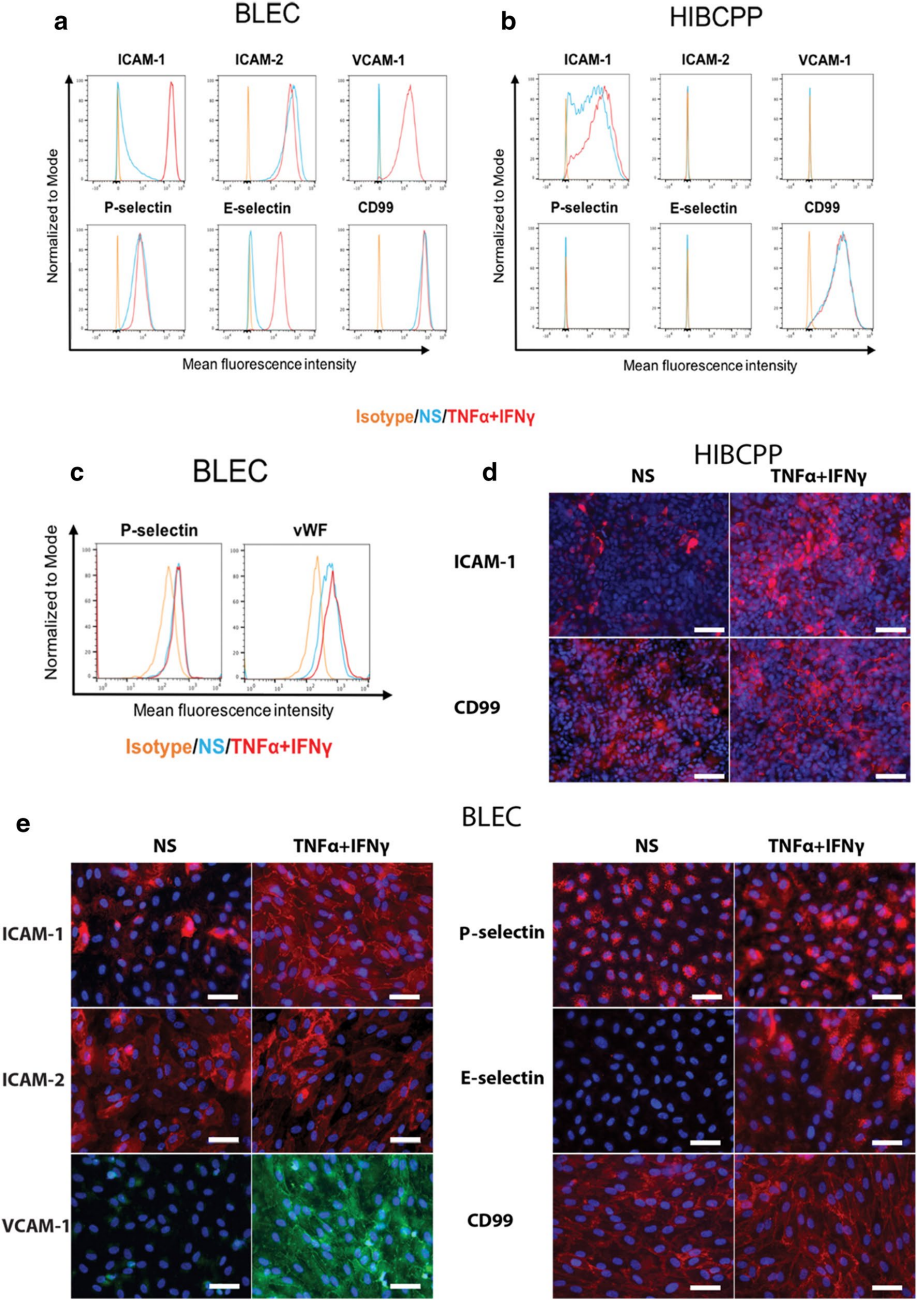
Adhesion molecule phenotype of BLECs and HIBCPP cells. Cell surface staining of BLECs (**a**) and HIBCPP cells (**b**) for the adhesion molecules ICAM-1, ICAM-2, VCAM-1, P-selectin, E-selectin and CD99 was analyzed by flow cytometry. **c** Cell surface staining of BLECs for the P-selectin and von Willebrand factor (vWF) was analyzed by flow cytometry. Isotype control, non-stimulated (NS) and 16 h pro-inflammatory cytokine-stimulated condition (1 ng/mL TNF-α + 20 IU/mL IFN-γ) are represented respectively in orange, blue and red in a histogram overlay. Immunofluorescence staining on BLECs (**e**) and HIBCPP cells (**d**) for ICAM-1 (red), ICAM-2 (red), VCAM-1 (green), P-selectin (red), E-selectin (red) and CD99 (red). Nuclei were stained with DAPI (blue). Each staining is representative of at least 3 independent experiment performed on 3 distinct filters. Both, NS and 1 ng/mL TNF-α + 20 IU/mL IFN-γ stimulated conditions are shown. Scale bar = 50 μm

We first asked if BLECs and HIBCPP cells display expression of adhesion molecules described on the BBB and BCSFB in vivo [3]. To this end, we performed flow cytometry analysis of non-stimulated (NS) or cytokine-stimulated (1 ng/mL TNF-α + 20 IU/mL IFN-γ) BLECs and HIBCPP cells for the adhesion molecules ICAM-1, ICAM-2, VCAM-1, P-selectin, E-selectin and CD99 as these molecules have been implied to mediate T-cell migration across the brain barriers. BLECs stained positive for ICAM-1, ICAM-2 and CD99 under NS conditions (Fig. 1a). After stimulation with pro-inflammatory cytokines, cell surface staining of ICAM-1 increased, while staining for ICAM-2 and CD99 did not change (Fig. 1a). To our surprise we found cell surface staining for P-selectin on NS and cytokine-stimulated BLECs, which was in part due to the release of P-selectin from Weibel-Palade bodies during preparation of single BLEC suspensions as shown by a similarly positive cell surface staining observed for von Willebrand factor (Fig. 1c). VCAM-1 and E-selectin could not be detected on non-stimulated BLECs and were found upregulated on BLECs after pro-inflammatory cytokine stimulation (Fig. 1a). Detection of these adhesion molecules in BLECs monolayers could be confirmed by performing immunofluorescence stainings on BLEC monolayers (Fig. 1e).

HIBCPP cells stained positive for ICAM-1 and CD99, and as expected not for ICAM-2, P- and E-selectin under both NS and cytokine-stimulated conditions (Fig. 1b, d). While surface staining for ICAM-1 on HIBCPP cells increased upon 16 h pro-inflammatory cytokine stimulation compared to NS condition, staining for CD99 remained unchanged. HIBCPP cells did not stain positive for VCAM-1 (Fig. 1b). Thus, BLECs show adhesion molecule expression and upregulation as observed in vivo, while HIBCPP lack expression of VCAM-1. Nevertheless, as HIBCPP cells stained positive for CD99 and ICAM-1, expressed by choroid plexus epithelial cells in vivo they are still a useful model to study T-cell migration across the BCSFB.

### Barrier characteristics of the human in vitro BBB and BCSFB models

**Fig. 2:**
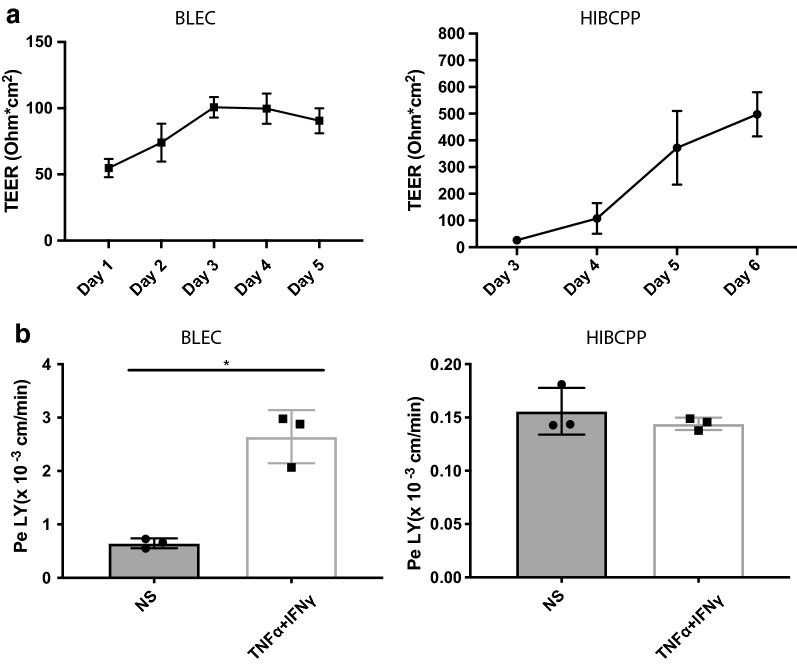
Barrier characteristics of BLECs and HIBCPP cells. **a** The time-dependent progression of the trans-endothelial or epithelial electrical resistance (TEER) of BLECs and HIBCPP cell monolayers were measured by Volt-Ohm-Meter. BLECs were seeded onto 0.4 μm pore size Costar Transwell^®^ filters and HIBCPP cells onto 5 μm pore size Millicell^®^ filters over a period of 6 days. Solid lines represent mean and error bars show ± SD. Data are representative data from at least 3 experiments each performed in triplicates. **b** Permeability for 0.45 kDa Lucifer Yellow (LY): BLECs were cultured onto 3 μm pore size Coster Transwell^®^ filters for 7 days as a monoculture and then co-culture with pericyte for 6 days and permeability was measured at day 13. HIBCPP cell were cultured onto 5 μm pore size Millicell^®^ filters and permeability was measured at day 6. Bars show the mean permeability coefficients Pe ± SD of diffused tracer across the BLECs or HIBCPP cell monolayer. Data are Representative data from at least 3 independent experiments with three filters per conditions. Statistical analysis: Student’s t-test p < 0.05*

We next analyzed and directly compared the barrier characteristics of BLECs and HIBCPP cell monolayers by determining transendothelial and transepithelial electrical resistance (TEER) and permeability to a small hydrophilic tracer of the in vitro BBB and BCSFB models. We first compared TEER values of BLEC and HIBCPP monolayers at the date of transmigration assays. We found that HIBCPP cells showed higher TEER values (497.7 ± 82.7 Ω × cm^2^) compared to BLECs (90.5 ± 9.5 Ω × cm^2^) (Fig. 2a) underscoring that, in their respective culture conditions, the BCSFB model is a tighter barrier than the BLEC model. This was confirmed when measuring the permeability of BLEC and HIBCPP monolayers for the small molecular tracer Lucifer Yellow (LY) with an average molecular weight of 0.45 kDa. In accordance to previous findings [47], BLECs cultured on 3 μm pore filter inserts showed very low permeability to LY (Pe_LY_ = 0.647 ± 0.091 × 10^−3^ cm/min). As expected, pro-inflammatory cytokine stimulation of BLECs significantly increased the permeability to LY (Pe_LY_ = 2.643 ± 0.499 × 10^−3^ cm/min) (Fig. 2b). In contrast, paracellular permeability of the HIBCPP monolayers to LY was fourfold lower than that of the BLEC monolayers (Pe_LY_ = 0.156 ± 0.022 × 10^−3^ cm/min) and was not affected by pro-inflammatory cytokine stimulation of the HIBCPP cells (Pe_LY_ = 0.144 ± 0.006 × 10^−3^ cm/min). Thus, both BLECs and HIBCPP cells establish functional BBB and BCSFB properties respectively, with the HIBCPP cells forming a significantly tighter barrier compared to BLECs as described in mouse models [29, 48, 49].

### Human CD4 Th cell subsets differ in their ability to cross the BBB and BCSFB

To explore if human Th cell subsets differ in their ability to cross the BBB and BCSFB we directly compared the migration of Th1, Th1*, Th2 and Th17 cells isolated from 3 different healthy donors across BLECs and HIBCPP cell monolayers, respectively.

**Fig. 3:**
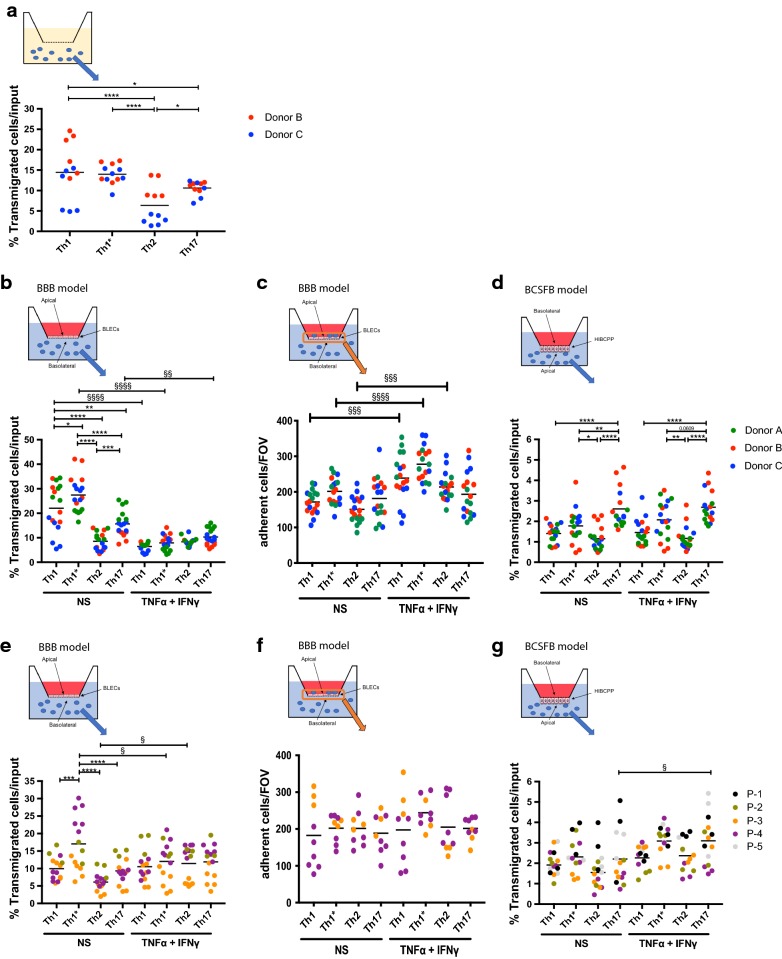
Transmigration assay across BLECs and HIBCPP cells. **a** Spontaneous T-cell migration over 2 h across laminin coated 5 μm pore size Millicell^®^ filters is shown. The graph shows the percentage of transmigrated T-cells of healthy donors B (red) and C (blue). Data are displayed as the mean on a superimposed scatter dot plot of 4 independent experiments; 2 experiments for each donor each in triplicates. Statistical analysis: two-way ANOVA followed by Sidak’s multiple comparison (p < 0.05*, p < 0.0001****). **b**, **d**, **e**, **g** CD4^+^ T-cell (Th1, Th1*, Th2 and Th17) migration rate across non-stimulated (NS) or 16 h pro-inflammatory cytokine-stimulated (1 ng/mL TNF-α + 20 IU/mL IFN-γ) BLECs and inverted HIBCPP cell monolayers was measured after 8 h transmigration assay. Percentages of transmigrated T cells from peripheral blood of three healthy donors (Donor A–C) and CSF of five MS patients (P-1–P-5) across BLECs (**b**, **e**) and inverted HIBCPP cell monolayer (**d**, **g**) are displayed. Data are shown as the mean on superimposed scatter dot plot of 5 or 6 independent experiments each performed in triplicates. Statistical analysis: two-way ANOVA followed by Tukey’s multiple comparison test within conditions (between subsets) (p < 0.05*, p < 0.01**, p < 0.001***, p < 0.0001****). Two-way ANOVA followed by Sidak’s multiple comparison test (NS versus stimulated condition) (p < 0.05^§^, p < 0.01^§§^, p < 0.0001^§§§§^). **c**, **f** The numbers of CD4^+^ T-cell (Th1, Th1*, Th2 and Th17) adherent to BLECs monolayer after transmigration assay were displayed. Data are shown as the mean on superimposed scatter dot plot of 3 or 6 independent experiments each performed in triplicates. Statistical analysis: two-way ANOVA followed by Sidak’s multiple comparison test (NS versus stimulated condition) (p < 0.001^§§§^, p < 0.0001^§§§§^). Cells used for the representation of the endothelium, the epithelium (HIBCPP cells) and T cells are adapted from Servier Medical Art (http://smart.servier.com/), licensed under a Creative Common Attribution 3.0 Generic License

To determine if the intrinsic motility of the different Th cell subsets differs in a significant manner we first compared the spontaneous migration of Th1, Th1*, Th2 and Th17 cells isolated from 2 different donors across laminin-coated filters for a period of 2 h. The spontaneous migration of Th1 and Th1* cells into the lower chamber was comparable and significantly higher when compared to Th17 and Th2 cells (Fig. 3a). Th2 cells showed the lowest intrinsic motility, which was significantly lower when compared to all other Th cell subsets.

To explore the ability of the different Th subsets to cross the BBB versus the BCSFB under inflammatory and non-inflammatory conditions we investigated their spontaneous migration across cytokine-stimulated and non-stimulated BLECs and HIBCPP monolayers. Under conditions of immune surveillance, e.g. in the absence of cytokine stimulation significantly higher fractions of Th1* cells, followed by Th1 cells crossed the BLEC monolayer over a period of 8 h when compared to Th17 and Th2 cells (Fig. 3b). This Th-cell subset specific migration behavior was observed for the Th cells from all 3 donors investigated and was different from their intrinsic migration behavior underscoring that different Th subsets have different abilities to cross the BBB under conditions of CNS immune surveillance. Interestingly, under inflammatory conditions the migration of all Th subsets, except for the Th2 subset, across the BLEC monolayer was found to be significantly reduced when compared to non-inflammatory conditions, and at the same time the migration rates for all Th subsets across the cytokine-stimulated BLEC monolayer were now comparable (Fig. 3b). This suggests that under inflammatory conditions the mechanisms controlling T-cell migration across the BBB change and apply in a similar fashion to all CD4^+^ T-cell subsets.

Since we found reduced numbers of Th cells to migrate across cytokine stimulated BLECs monolayers, we asked if Th cells adhere better to the inflamed BLECs monolayer in our experimental setting. To test this, we fixed and stained BLECs monolayer after the transmigration assay and counted firmly adhered Th cells on BLECs. We found that significantly higher numbers of Th1, Th1* and Th2 cells adhere to cytokine stimulated BLECs compare to non-stimulated BLECs (Fig. 3c). This suggests that Th cells from the peripheral blood of healthy donors adhere better to inflamed BLECs in our experimental setting and in part explain the reduction of transmigrated Th cells across cytokine stimulated BLECs.

We next investigated the ability of the identical Th subsets from the same 3 healthy donors to migrate across the in vitro BCSFB model. In general, migration of the Th cell subsets from the basolateral (choroid plexus stroma-facing) side to the apical (CSF-facing) side of the HIBCPP monolayers was about tenfold lower when compared to their migration across the BLEC monolayers during the same period of 8 h. Also, we did not observe any significant difference in the migration rates of Th cells across HIBCPP monolayers in the absence or presence of inflammatory stimulation, suggesting that molecular cues required for Th cell migration across the BCSFB do not change upon cytokine stimulation. The Th17 cells and to a lesser degree the Th1* cells from all 3 healthy donors were found to cross the HIBCPP monolayer in significantly higher fractions, when compared to Th1 and Th2 cells (Fig. 3d). These observations suggest that Th17 and Th1* cells can preferentially use the BCSFB for CNS entry via the CSF filled ventricle and furthermore show that different Th cell subsets can explore different brain barriers for their preferential entry into the CNS.

### CSF-derived Th cell subsets from MS patients do not display enhanced migration across the brain barriers

We next asked if T-cell subsets isolated from the CSF of MS patients and thus experienced in migration across brain barriers show enhanced abilities to cross the BBB or BCSFB. To this end, we used expanded CD4^+^ T cells isolated from the CSF of 5 MS patients and studied their migration across the BBB and BCSFB in the same manner as the migration of circulating Th cells from healthy donors. In general, CSF derived Th cell subsets from MS patients did not show significantly enhanced migration across the BBB and BCSFB, when compared to circulating Th cell subsets from healthy donors (Fig. 3e, g). Although the migration rate of Th-cell subsets derived from CSF of MS patients was variable dependent on patients, CSF derived Th1* cells crossed the BLECs monolayers in higher numbers, when compared to Th1, Th17 and Th2 cells in the absence of inflammatory stimuli (Fig. 3e). As already observed for the Th-cell subsets isolated from peripheral blood of healthy donors, all CSF derived Th cell subsets showed a similar capacity to cross the BLEC monolayer under neuroinflammatory conditions. Unlike Th cells derived from peripheral blood of healthy donors, only CSF derived Th 1* cells showed reduced migration rate across inflamed BLECs compared to non-inflammatory BLECs. At the same time, we found that Th cells derived from CSF of MS patients showed no significant difference of adhesion to BLECs upon inflammation (Fig. 3f). When studying the migration of CSF derived Th cell subsets across the BCSFB, we observed the trend that CSF derived Th17 and Th1* cells from MS patients crossed the HIBCPP monolayer in higher fractions under both inflammatory and non-inflammatory conditions when compared to Th1 and Th2 cells, though it was not significant because of higher variability depending on the patient (Fig. 3g). These observations suggest that barrier crossing experienced Th cell subsets derived from the CSF of MS patients do not show a significantly increased ability to cross the brain barriers, when compared to Th-cell subsets isolated from the peripheral blood of healthy donors. Our study therefore underscores the active role of the brain barriers in controling the migration of different Th cell subsets into the CNS during inflammatory and non-inflammatory conditions.

### Molecular mechanisms mediating CD4^+^ T cells across the BCSFB

**Fig. 4:**
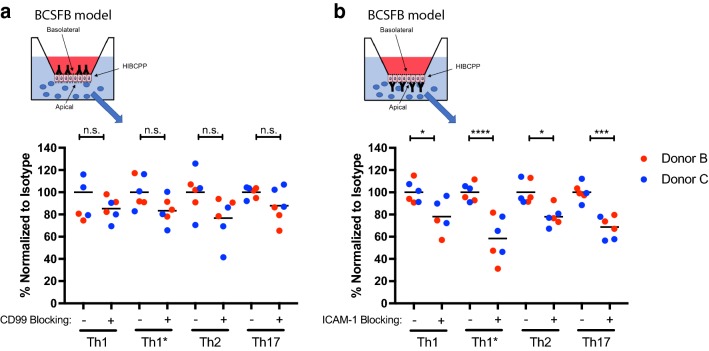
Molecular mechanisms mediating CD4^+^ T-cell migration across HIBCPP from choroid plexus stroma side to CSF side. Percentage of transmigrated T-cells from peripheral blood of healthy donors B and C across 16 h pro-inflammatory cytokine-stimulated (1 ng/mL TNF-α + 20 IU/mL IFN-γ) inverted HIBCPP cells monolayer pretreated with either anti-human CD99 blocking antibody (20 μg/mL) (**a**), anti-human ICAM-1 blocking antibody (10 μg/mL) (**b**) or isotype control antibody are shown. CD4^+^ T-cells (Th1, Th1*, Th2 and Th17) were allowed to migrate across inverted HIBCPP cells monolayer for 8 h and migrated cells were collected and counted. Results are standardized to isotype control (100%). Data are shown as the mean on superimposed scatter dot plot of 2 independent experiments each performed in triplicates. Statistical analysis: two-way ANOVA followed by Tukey’s multiple comparison test (p < 0.05*, p < 0.001***, p < 0.0001****). Cells used for the representation of the endothelium, the epithelium (HIBCPP cells) and T cells are adapted from Servier Medical Art (http://smart.servier.com/), licensed under a Creative Common Attribution 3.0 Generic License

We have previously shown that all CD4^+^ Th-cell subsets use ICAM-1 and CD99, but not platelet endothelial cell adhesion molecule (PECAM)-1 to cross cytokine stimulated BLEC monolayers under static conditions [32]. Here we asked if ICAM-1 and CD99 also mediate Th-cell migration across the BCSFB from the choroid plexus stroma to the CSF-facing side. Investigating the migration of Th cell subsets from peripheral blood of healthy donors across HIBCPP monolayers, we found that antibody-mediated blocking of epithelial ICAM-1 significantly reduced the migration of all human Th-cell subsets across TNF-α/IFN-γ-stimulated HIBCPP monolayers (Fig. 4b). We also found a trend towards reduced numbers of human CD4^+^ T-cell subsets migrating across HIBCPP in the presence of CD99 blocking antibodies (Fig. 4a). To confirm the efficacy of the CD99 blocking antibody, we compared side-by-side the effect of antibody mediated blocking of CD99 on Th1-cell migration across the BLECs and HIBCPP monolayers. This confirmed our previous observations on the role of CD99 in Th1 migration across BLECs [32] (data not shown) and thus efficacy of CD99 function blocking of our antibody.

At the same time, we observed that blocking ICAM-1 comparably reduced the migration of all Th subsets across HIBCPP monolayers, suggesting that ICAM-1 is required for the migration of all Th cell subsets across the BCSFB. As ICAM-1 is only expressed on the apical (CSF-facing) side of HIBCPP cells and therefore not directly available at the basolateral side for T-cell migration across the BCSFB, these results suggest that the Th cells may require epithelial ICAM-1 at the last step of the transepithelial diapedesis cascade.

### CD4^+^ Th cells can cross the BCSFB from the CSF to the choroid plexus stroma side

**Fig. 5:**
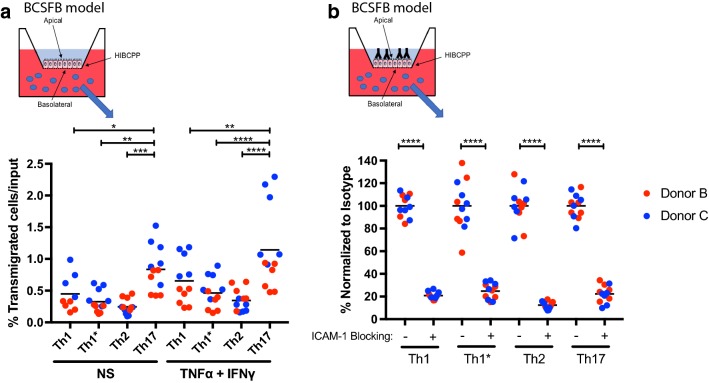
CD4^+^ T-cell migration across HIBCPP from CSF side to choroid plexus stroma side. **a** CD4^+^ T-cell (Th1, Th1*, Th2 and Th17) migration rate across non-stimulated (NS) or 16 h pro-inflammatory cytokine-stimulated (1 ng/mL TNF-α + 20 IU/mL IFN-γ) standard HIBCPP cell monolayers was measured after 8 h transmigration assay. Percentages of transmigrated T cells from peripheral blood of two healthy donors (Donor B and C) across standard HIBCPP cell monolayer are displayed. Data are shown as the mean on superimposed scatter dot plot of 4 independent experiments each performed in triplicates. Statistical analysis: two-way ANOVA followed by Tukey’s multiple comparison test within conditions (between subsets) (p < 0.05*, p < 0.01**, p < 0.001***, p < 0.0001****). **b** Percentage of transmigrated T-cells from peripheral blood of healthy donors B and C across 16 h pro-inflammatory cytokine-stimulated (1 ng/mL TNF-α + 20 IU/mL IFN-γ) standard HIBCPP cells monolayer pretreated with either anti-human ICAM-1 blocking antibody (10 μg/mL) or isotype control antibody are shown. CD4^+^ T-cells (Th1, Th1*, Th2 and Th17) were allowed to migrate across standard HIBCPP cells monolayer for 8 h and migrated cells were collected and counted. Results are standardized to isotype control (100%). Data are shown as the mean on superimposed scatter dot plot of 4 independent experiments each performed in triplicates. Statistical analysis: two-way ANOVA followed by Tukey’s multiple comparison test. (p < 0.0001****). Cells used for the representation of the endothelium, the epithelium (HIBCPP cells) and T cells are adapted from Servier Medical Art (http://smart.servier.com/), licensed under a Creative Common Attribution 3.0 Generic License

Since a recent study has proposed that T cells may exit the CNS via the choroid plexus [50], we next investigated if the different Th cell subsets can cross the HIBCPP monolayers from the apical to the basolateral side, thus mimicking their migration from the CSF side into the choroid plexus stroma. To this end, we used a standard culture system of HIBCPP cells and first tested the barrier characteristics of HIBCPP monolayers in this culture system. We found that while the TEER of HIBCPP monolayers was significantly higher in the standard culture system (640.3 ± 49.3 Ω × cm^2^) when compared to the inverted culture system (497.7 ± 82.7 Ω × cm^2^), the permeability for Lucifer yellow was comparable for both systems (Pe_LY_ = 0.144 ± 0.006 × 10^−3^ cm/min, and 0.156 ± 0.022 × 10^−3^ cm/min, respectively, for the standard and inverted culture system). We next investigated the migration of the different Th-cell subsets derived from the peripheral blood of healthy donors across the HIBCPP monolayers over 8 h in the presence or absence of prior proinflammatory cytokine-stimulation (1 ng/mL TNF-α + 20 IU/mL IFN-γ for 16 h). In general we observed that the migration of the different Th-cell subsets from the apical to the basolateral site of HIBCPP cell monolayers was significantly lower when compared to their migration from the basolateral to apical site of HIBCPP monolayers (Figs. 3d, 5a). Th17 cells showed a significantly increased ability to cross the HIBCPP monolayers from the apical to basolateral side, when compared to Th1, Th1* and Th2 cells under both, non-stimulated and 16 h pro-inflammatory cytokine-stimulated conditions (Fig. 5a), exactly as previously observed when comparing the migration of the same Th cell subsets across HIBCPP monolayers from the basolateral to apical side (Fig. 3d). These observations underscore that low numbers of CSF derived CD4^+^ T cells may be able to leave the CNS via the choroid plexus.

Therefore we finally asked if apically expressed epithelial ICAM-1 mediates Th cell migration from the apical to the basolateral site of the BCSFB. To this end, we pre-incubated standard culture HIBCPP cell monolayers with anti-human ICAM-1 blocking antibodies and subsequently investigated the migration of Th-cell subsets derived from the blood of healthy donors across the HIBCPP monolayer. We found that blocking of epithelial ICAM-1 significantly reduced the migration of all CD4^+^ Th cell subsets across the standard culture HIBCPP cell monolayer to the same degree (Fig. 5b). Importantly, the percentage of T-cell migration across the HIBCPP cell monolayers was reduced fourfold more in the standard culture (Fig. 5b) when compared to T-cell migration across the inverted culture HIBCPP cell monolayers (Fig. 3c). This supports the notion that lack of availability of apical ICAM-1 interferes with the first adhesive step in T-cell migration from the apical to abluminal site of the BCSFB.

## Discussion

Three main routes for CD4^+^ T-cell entry into the CNS have been identified to date [1, 51]: from the blood via the choroid plexus stroma across the choroid plexus epithelium (BCSFB) into the CSF filled ventricles, from blood to the CSF filled subarachnoid space at the brain or spinal cord surface, and from blood to parenchymal perivascular spaces at the level of post capillary venules (BBB) [51]. Recent advances of in vivo live cell imaging technique allow us to investigate dynamic interactions of immune cells and CNS barrier-forming endothelial cells [52], however, because of the preferential accessibility at the brain and spinal cord surface the main focus of these studies has been immune cell interaction with leptomeningeal microvessels. Thus, studying the active role of the BBB and BCSFB in regulating the migration of different T-cell subsets into the CNS in vivo in its entire complexity is still difficult. A further limitation may be that molecular mechanisms identified for immune cell crossing of the BBB and BCSFB in animal models may not fully translate to the situation in humans.

To improve our understanding on the cellular and molecular mechanisms established at the endothelial BBB and the epithelial BCSFB that control the entry of different Th cell subsets into the CNS we here employed human in vitro models for the BBB and the BCSFB and human CD4^+^ T-cell subsets isolated from healthy donors and MS patients. We side-by-side compared the ability of Th1, Th2, Th17 and Th1* cells of the same donors in crossing the BBB and the BCSFB. Our data provide in vitro evidence that human Th1 and Th1* cells preferentially cross the BBB under non-inflammatory conditions, while Th17 cells showed increased ability compared to the other Th subsets from the same donor to cross the BCSFB irrespective of the inflammatory state. Notably, this preferential migratory behavior of Th17 cells was also observed for CNS entry experienced Th17 cells isolated from the CSF of MS patients, suggesting that the BCSFB at the level of the choroid plexus actively controls T-cell entry into the CNS.

There is indeed increasing evidence that the choroid plexus plays an important role for CNS immunity and immune mediated disorders such as MS. Comparative transcriptome analyses from the choroid plexus of healthy and EAE mice showed significant increase in the expression of genes encoding for adhesion molecules, T-cell activation markers as well as important chemokines and cytokines [53, 54]. These observations attribute a relevant role to the choroid plexus for controlling T-cell migration into the CNS during immune surveillance and neuroinflammatory conditions [53, 54]. CNS T-cell entry via the choroid plexus implies a multistep process, where initially T cells have to extravasate via the choroidal microvessels, which lack a BBB, reach the choroid plexus stroma and in a second step cross the BCSFB, formed by choroid plexus epithelial cells, to reach CSF filled ventricular spaces of the CNS. A previous report showed that circulating T cells extravasate in a P-selectin-dependent manner across fenestrated capillaries to reach the choroid plexus stroma [55]. However, direct evidence showing how T cells located in the choroid plexus stroma enter the CNS is limited [49]. Although we here observed that the transmigration rate of all Th cell subsets across the HIBCPP monolayer was extremely low compared to BLECs, we still observed that all CD4^+^ Th subsets could cross the HIBCPP monolayers under both, non-inflammatory and inflammatory conditions (Fig. 3b and c). Our data thus further support the notion that the choroid plexus is a potential T-cell entry site for human CD4^+^ T cells under both, immune surveillance and neuroinflammation. Notably, Th17 cells showed an increased ability to cross the BCSFB compared to other Th subsets from the very same donor, which corresponds to our previous in vivo findings in a mouse model of EAE which showed that CCR6^+^ Th17 cells may preferentially enter the brain via the BCSFB to induce EAE [19]. We also found that significantly higher numbers of Th1* cells migrate across the BLEC monolayers under non-inflammatory conditions compared to the Th subsets of the very same donors (Fig. 3b). Th1* cells have been found in MS lesions [12] and are more abundant in the CSF compared to blood in MS patients [9]. Potential pathogenicity of Th1* cells is further supported by their pro-inflammatory phenotype by secreting IFN-γ, IL-17 and GM-CSF [9, 12] and their identification as auto-proliferating CD4^+^ T cells, enriched in brain-homing cells [15]. Accumulation of Th1* cell subsets in peripheral blood is also observed in natalizumab-treated relapse-free RRMS patients, but not in natalizumab-treated patients during a relapse [9, 15]. In fact, the latter study provided evidence that Th1* cells show enhanced migration to the brain or CSF compartments during MS relapses. Combined with our present findings that there is no difference in the ability of different Th cell subsets from the very same donor to cross the BBB under inflammatory conditions, one may speculate that the migration of Th1* cells across the BBB is rather relevant in the initiation phase of an MS relapse. Taken together identification of the molecular mechanisms that mediate enhanced migration of Th1* cells and Th17 cells across the non-inflamed BBB and BCSFB, respectively, may open avenues to specifically block CNS entry of pathogenic T-cell subsets and thus preventing MS relapses while still allowing for Th cell entry required for CNS immune surveillance.

While the role of adhesion molecules on BBB endothelium has been studied intensively (reviewed in [3]), less is known about adhesion molecule expression at the BCSFB-forming epithelium. Data from rodent models show that ICAM-1 and VCAM-1 are constitutively and functionally expressed at the BSCFB on the apical side of choroid plexus epithelial cells and upregulated during EAE [56, 57]. However, it still remains unclear whether these adhesion molecules, with their exclusive polarized location on the apical (CSF-facing) side of the choroid plexus epithelial cells, play a relevant role in T-cell migration across the BCSFB from choroid plexus stroma to the ventricular space. We here show that antibody-mediated blockade of epithelial ICAM-1 had a slightly however significantly reduced the migration of all Th subsets across the BCSFB from the basolateral to apical side (Fig. 4b). It has been previously shown that apically expressed ICAM-1 in intestinal epithelium contributes to finalization of the migration of neutrophils across this barrier and their sustained adhesion to the apical surface of this epithelium leading to activation of epithelial Akt and β-catenin signaling and wound healing [58, 59]. Thus, apical ICAM-1 at the BCSFB may fulfill a similar role in guiding the final steps of T cells having crossed the epithelial barrier by adhesive interactions. Th cells expressing the ICAM-1 ligand LFA-1 might use ICAM-1 on the apical side of the BCSFB as a molecular anchor to firmly adhere on the apical side of the choroid plexus epithelium and to complete their transmigration from the choroid plexus stroma to the CSF ventricle spaces. Not too surprisingly we observed that function blocking of ICAM-1 almost abolished the low rate of migration of the different CD4^+^ T cells observed across the choroid plexus epithelium from the CSF to the choroid plexus stroma side (Fig. 5b) underscoring the prominent role of ICAM-1 in mediating T-cell adhesion and potentially migration across the BCSFB from the apical to the basolateral side.

We have previously shown that in addition to ICAM-1, CD99 mediates the migrations of different human CD4^+^ T cells across the BBB endothelium in vitro [32]. Here we provide additional evidence that different human CD4^+^ T cells also use ICAM-1 and possibly CD99 when crossing the BCSFB. It is worth to emphasize that function blocking of ICAM-1 or CD99 equally affected the migration of all CD4^+^ T-cell subsets from the same donors. Furthermore, ICAM-1 was shown to mediate the migration of other immune cell subsets (e.g. CD8^+^ T cells [60], B cells [61], polymorphonuclear leukocytes [62], monocytes [63]) across the BBB. Taken together this underscores an important role for ICAM-1 in CNS entry of innate and adaptive immune cells maintaining CNS immunity. In fact, Efalizumab, a monoclonal antibody targeting LFA-1, when previously used for the treatment of psoriasis patients, was shown to cause high rates of PML [64, 65] and was thus withdrawn from the market. In contrast to ICAM-1, less is known about the role of CD99 in mediating immune cell migration into the CNS. CD99 was shown to regulate the migration of monocytes [66] and CD4^+^ T cells [32] across in vitro models of the BBB. Here we also found that blocking CD99 reduced the migration of all CD4^+^ T-cell subsets across the choroid plexus epithelium with to a comparable degree. These observations further underscore the active role of the BCSFB in controlling Th cell subset entry into the CNS.

Precise involvement of chemokines or lipid mediators binding to G-protein coupled receptors (GPCRs) on the different Th cell subsets in their respective migration across the BBB and BCSFB remain to be explored. Engagement of GPCRs on the T cells induces inside-out activation of cell surface expressed adhesion molecules of the integrin family allowing for the sustained firm arrest of T cells on the BBB [3]. Observations made by us and others showed that while GPCR signaling is not required for post-arrest crawling of effector Th cells on vascular endothelial cells under physiological flow, it is required for their diapedesis across the endothelial monolayer [60, 67]. Preliminary observations made with the Th cell subsets used in the present study confirm involvement of GPCR signaling in the diapedesis of all Th subsets across BLEC monolayers. Furthermore, our previous studies have shown that addition of exogenous CXCL12 to the CSF side increased T-cell migration across HIBCPP monolayers [31, 68, 69]. These data support the additional role for GPCR signaling in the migration of effector Th cell subsets across the BBB and BCSFB. The precise nature of the GPCR ligands involved in T cell migration across the BBB and BCSFB during immune surveillance and neuroinflammation remain to be explored.

Since the capillaries at the choroid plexus are fenestrated, and thus lack a BBB, immune cells located in the choroid plexus stroma are additionally exposed to peripheral stimuli [50]. Indeed, magnetic resonance imaging (MRI) using iron oxide magnetic nanoparticles combined with ex vivo histological analysis showed that the iron oxide particles were taken up by immune cells present in the choroid plexus stroma in mouse models of EAE or following intraperitoneal administration of lipopolysaccharide [70–72]. These observations suggest that the choroid plexus is involved in CNS immunity and plays and active role already during the early inflammatory response. However, it is not clear if immune cells present in the choroid plexus stroma have previously entered from the blood stream of the CNS. To this end, we invested the migration of different Th cell subsets across the HIBCPP monolayers from the CSF side to choroid plexus stroma side. Although their migration rates were very low when compared to their migration from the basolateral to apical side, we here provide in vitro evidence that all investigated Th subsets could also migrate across the choroid plexus epithelium from CSF side to choroid plexus stroma side. Our findings are thus in accordance to previous observations in mouse models that showed that intracerebroventricularly injected activated Th1 cells could reach the choroid plexus stroma in an ICAM-1 dependent manner [50].

## Conclusion

In conclusion, we here show that human Th cell subsets can cross in vitro models of the human BBB endothelium and BCSFB epithelium. Our study underscores that the brain barriers hereby actively control the migration of the different Th cell subsets during inflammatory and non-inflammatory conditions. We also show that different human Th cells use different mechanisms to cross the BBB versus the BCSFB during immune surveillance and neuroinflammatory conditions confirming previous observations in animal models [18–21]. Therefore, understanding the different molecular mechanisms mediating the migration of different Th cell subsets across the BBB and the BCSFB into the CNS during immune surveillance and neuroinflammation bears the hope for the development of therapies preferentially blocking CNS entry of pathogenic T cells while leaving CNS entry of those required for CNS immune surveillance largely unaffected.

## Data Availability

All data generated or analysed during this study are included in this published article.

## Abbreviations

BBB: blood–brain barrier
BCSFB: blood cerebrospinal fluid barrier
BLECs: brain-like endothelial cells
ChP: choroid plexus
CNS: central nervous system
CSF: cerebrospinal fluid
EAE: experimental autoimmune encephalitis
FBS: fetal bovine serum
GWASs: genome-wide association studies
HIBCPP: human choroid plexus papilloma cell line
ICAM-1: intercellular adhesion molecule-1
LFA-1: lymphocyte function-associated antigen 1
MRI: magnetic resonance imaging
MS: multiple sclerosis
NS: non-stimulated
Pe: permeability coefficient
PECAM-1: platelet endothelial cell adhesion molecule
PML: progressive multifocal leukoencephalopathy
RRMS: relapsing–remitting multiple sclerosis
RT: room temperature
TEER: transendothelial or transepithelial electrical resistance
Th cells: T helper cells
LY: Lucifer Yellow
VCAM-1: vascular cell adhesion molecule-1
vWF: von Willebrand factor
